# Does periodontitis influence the risk of COVID‐19? A scoping review

**DOI:** 10.1002/cre2.584

**Published:** 2022-05-17

**Authors:** Arunibha Ghosh, Betsy Joseph, Sukumaran Anil

**Affiliations:** ^1^ SN Pradhan Centre for Neurosciences University of Calcutta Kolkata India; ^2^ Department of Periodontics, Saveetha Dental College and Hospital Saveetha Institute of Medical and Technical Sciences Chennai India; ^3^ Hamad Medical Corporation Doha Qatar; ^4^ College of Dental Medicine Qatar University Doha Qatar

**Keywords:** cytokines, oral–lung axis, periodontopathogens, SARS‐CoV‐2

## Abstract

**Objective:**

Research has shown that the novel severe acute respiratory syndrome coronavirus‐2 (SARS‐CoV‐2) significantly influences the oral microbiome to expedite systemic diseases by invading harmful oral pathogens near and distant organs. To identify, explore, and map the possible mechanisms underlying periodontitis in severe coronavirus disease 2019 (COVID‐19) cases.

**Material and Methods:**

Relevant articles published from December 2019 to February 2022 were identified and screened using keywords and inclusion criteria from various databases.

**Results:**

This review sheds light on multiple pathways of periodontitis, the spread of periodontal infection and microbial metabolites to the lungs, and the dysregulated immune system with elevated cytokines, reactive oxygen species generation, nuclear DNA damage, and senescence, which have the potential to promote stronger viral attachment to host cells and the onset of COVID‐19 manifestation with increased severity and risk of mortality. In addition, the cytokine connection to SARS‐CoV‐2, T‐cell responses against periodontitis, its connection with COVID‐19, the role of host factors, and periodontal therapy have been discussed.

**Conclusions:**

The relationship between COVID‐19 and periodontitis needs further investigation along with the development of alternative therapies to prevent periodontitis for better management and control of COVID‐19.

## 1 INTRODUCTION

Coronavirus disease 2019 (COVID‐19) is a severe acute respiratory infection caused by severe acute respiratory syndrome coronavirus‐2 (SARS‐CoV‐2) that emerged in Wuhan, Hubei, China, with a subsequent global spread (Ren et al., 2020; Zhu et al., 2020). The most common symptoms of COVID‐19 are fever, cough, and fatigue, with the occurrence of other symptoms, such as sputum production, headache, hemoptysis, diarrhea, dyspnea, and lymphopenia (Q. Li, Guan, et al., 2020; Rothan & Byrareddy, 2020). In severe cases, the COVID‐19 infections can cause pneumonia, kidney failure, and even death, particularly in individuals with other chronic health conditions. The duration from the onset of COVID‐19 symptoms to death ranges from 6 to 41 days, with a median of 14 days (W. Wang, Tang, et al., 2020). In addition to acute respiratory distress and pneumonia, SARS‐CoV‐2 is associated with various systemic symptoms, including gastrointestinal (GI) disturbances and impairment in the function of the central nervous system (Y. C. Li, Bai, et al., 2020; Mao et al., 2020). SARS‐CoV‐2 may induce alterations in the central nervous system without directly crossing the blood–brain barrier, instead of synthesizing inflammatory cytokines, created a “cytokine storm,” which triggers neurological dysfunction (J. Wang, Jiang, et al., 2020). Variations in the symptoms and classical signs of COVID‐19 have been reported as new variants of the SARS‐CoV‐2 clan.

The severe clinical course of the COVID‐19 infection has been linked to chronic disorders, such as cardiovascular disease, hypertension, diabetes mellitus, obesity, and chronic renal disease (Pfützner et al., 2020; Rapp et al., 2021). Additionally, SARS‐CoV‐2 infection results in dysgeusia in patients, as well as oral lesions and cutaneous manifestations of COVID‐19, due to the presence of angiotensin‐converting enzyme‐2 (ACE2) receptors on the oral mucosa, with a higher density on the dorsum of the tongue and salivary glands than on the buccal or palate mucosa (Iranmanesh et al., 2021; H. Xu, Zhong, et al., 2020). Poor oral health has been associated with the activation of several systemic diseases, including diabetes, obesity, atherosclerotic heart disease, Alzheimer's disease, and disease‐related consequences (Jepsen et al., 2015; Wu & Nakanishi, 2014). Additionally, lung infection may occur as a result of oral pathogen colonization of the lower respiratory tract or modification of mucosal surfaces, which accelerates the senescence of the lung epithelium, thus producing a favorable environment for severe SARS‐CoV‐2 infection (Aquino‐Martinez & Hernandez‐Vigueras, 2021; Botros et al., 2020). Periodontal disease has been linked to an increased risk of acquiring severe COVID‐19 infection, hospitalization, and mortality (Anand et al., 2021; Gupta et al., 2022; Marouf et al., 2021). Hence, this scoping review aimed to focus on the probable mechanisms underlying COVID‐19 and periodontitis.

## 2 METHODOLOGY

The Preferred Reporting Items for Systematic Reviews and Meta‐Analyses extension for scoping reviews checklist was used to perform this review (Tricco et al., 2018). The study protocol was designed before the study was performed following a preliminary review of the previous literature (Colquhoun et al., 2014; Peters et al., 2015). The primary question of the scoping review was “Does periodontitis affect the risk of COVID‐19?” This review aimed to explore and map the existing literature to determine whether periodontitis is associated with COVID‐19.

## 3 ELIGIBILITY CRITERIA

Articles published between December 2019 and February 2022 were screened for relevance by using the following PCC framework: Population: the presence of periodontitis; Concept: clinical studies; Context: association with COVID‐19. Clinical studies reporting periodontitis in patients with COVID‐19 were included in this scoping review. In contrast, commentaries, review articles, case reports, in vitro studies, editorials, letters to the editor, conference papers, consensus papers, and questionnaire studies, were eliminated.

## 4 SEARCH STRATEGY

Prominent literature databases, such as MEDLINE/PubMed, the Cochrane Library, Embase, Scopus, and Google Scholar, were extensively searched on February 28, 2022. The search was conducted based on the two main concepts (periodontitis and COVID‐19) of the research question. Articles that contained the MeSH terms, keywords, and other free terms related to “Periodontitis,” “Periodontopathogens,” “SARS‐CoV‐2,” “cytokines,” “cytokine storm,” “oral‐lung axis,” “senescence,” “dysbiosis,” or “COVID‐19,” were included in the initial screening. Additionally, references to relevant studies and manual searches were performed for other potentially appropriate publications.

A total of 480 articles were found in the preliminary search, of which 283 were excluded during the title and abstract screening based on the eligibility criteria. Duplicate articles were excluded with the help of a citation/reference manager (EndNote version 9). Two reviewers examined the remaining 197 articles in full length based on the inclusion and exclusion criteria. In case of disagreement, a third reviewer was contacted, who resolved the differences through discussion, and a final consensus was reached to include five studies. Table 1 shows the clinical studies included.

**Table 1:** Recent clinical studies that map the existing literature on whether the presence of PD is associated with COVID‐19

Author/country	Objective	Study design/sample size	Parameters	Conclusion
Gupta et al. (2022)/India	To conduct a clinical assessment of the relationship between periodontitis and COVID‐19‐related outcomes.	Case–control/84	Patient demographics, medical history, blood parameters, periodontal clinical examination, and aMMP‐8.	Periodontitis appears to be associated with adverse COVID‐19 outcomes. However, due to the constraints of this investigative process, the exact correlation may be difficult to confirm.
Anand et al. (2021)/India	Examine the relationship between periodontitis, inadequate oral hygiene, and COVID‐19.	Case–control/150	Periodontal parameters such as plaque scores, calculus scores, tooth mobility, gingival bleeding, probing depth, recession, and clinical attachment level were clinically examined.	There was a strong correlation between the severity of periodontitis and COVID‐19. Additionally, gingival bleeding and plaque accumulation were more frequent in patients with COVID‐19.
Gomes et al. (2021)/Brazil	This study aimed to determine the presence of SARS CoV‐2 RNA in the dental biofilms of symptomatic patients who tested positive for the virus in nasopharyngeal and oropharyngeal samples.	Observational/70	Dental BIO samples were collected and analyzed using RT‐qPCR to determine the virus's presence.	Dental biofilms from symptomatic patients with COVID‐19 contain SARS CoV‐2 RNA and may act as a reservoir for COVID‐19 transmission.
Marouf et al. (2021)/Qatar	To ascertain the relationship between periodontitis and COVID‐19 problems.	Case–control/568	Periodontal radiographic findings; BMI; D‐dimer, CRP, HbA1c, Vitamin D, white blood cells (WBC), and lymphocytes were also collected	Periodontitis was associated with an increased risk of ICU admission, assisted ventilation, and death in patients with COVID‐19, as well as with elevated blood levels of biomarkers associated with poor disease outcomes.
Larvin et al. (2020)/UK	The effect of PD on mortality in patients with COVID‐19	Case–control/13253	Self‐reported oral health indicators of periodontitis.	Inadequate evidence exists to establish a relationship between periodontal disease and COVID‐19 infection. A statistically significant increase in mortality was reported in COVID‐19 patients with periodontal disease.

Abbreviations: aMMP, activated matrix metalloproteinases; BIO, biofilm; BMI, body mass index; CRP, C‐reactive protein; COVID‐19, coronavirus disease 2019; HbA1c, hemoglobin A1c; ICU, intensive care unit; PD, periodontal disease; RT‐qPCR, real‐time quantitative polymerase chain reaction; SARS CoV‐2, severe acute respiratory syndrome coronavirus‐2; WBC, white blood cell.

## 5 DATA CHARTING AND ITEMS

The relevant data were selected and entered into an Excel spreadsheet by two reviewers, similar to the study selection. A third reviewer was involved if required. The author's name, year of publication, country, the objective of the study, study design, samples assessed, results, and conclusions were charted for all included studies.

## 6 SYNTHESIS OF RESULT

The data obtained through data extraction were used for the qualitative synthesis of the results. The results are presented in the next section.

## 7 RESULTS

Of the 480 articles identified during the preliminary search, a total of five studies matched the eligibility criteria of this scoping review; three were case–control studies (Anand et al., 2021; Larvin et al., 2020; Marouf et al., 2021), whereas the other two were cross‐sectional studies (Gomes et al., 2021; Gupta et al., 2022). The studies have been reported in India (Anand et al., 2021; Gupta et al., 2022), Brazil (Gomes et al., 2021), the United Kingdom (Larvin et al., 2020), and Qatar (Marouf et al., 2021) between 2020 and 2022. The sample sizes ranged from 70 (Gomes et al., 2021) to 13,253 (Larvin et al., 2020). These studies attempted to further answer questions related to the impact of periodontal disease on hospital admission and mortality during COVID‐19 (Larvin et al., 2020), association between periodontitis and poor oral hygiene with that of COVID‐19 (Anand et al., 2021), presence of SARS‐CoV‐ 2 RNA in the dental biofilm of symptomatic COVID‐19 patients (Gomes et al., 2021), association between periodontitis and COVID‐19‐related outcomes (Gupta et al., 2022), and association between periodontitis and COVID‐19 complications (Marouf et al., 2021).

Various parameters were used to answer these research questions, such as self‐reported oral health indicators of periodontal disease (Larvin et al., 2020); detailed periodontal examination such as plaque scores, calculus scores, tooth mobility, gingival bleeding, probing depth, recession, and clinical attachment level (Anand et al., 2021); real‐time quantitative polymerase chain reaction of dental biofilm samples (Gomes et al., 2021); patient demographics, medical history, blood parameters, clinical periodontal examination, and activated matrix metalloproteinases‐8 (Gupta et al., 2022); periodontal radiographic findings; body mass index; D‐dimer levels; C‐reactive protein levels; hemoglobin A1c levels; vitamin D levels; white blood cell counts; and lymphocyte counts (Marouf et al., 2021).

The results of this scoping review revealed heterogeneous outcomes. While a case–control study found that the risk of COVID‐19 infection in participants with painful or bleeding gingiva and mobile teeth compared to controls was not increased (odds ratio [OR]: 1.10, 95% confidence interval [CI]: 0.72–1.69; OR: 1.15, 95% CI: 0.84–1.59) (Larvin et al., 2020), another case–control study revealed significant associations of various periodontal parameters such as the mean plaque scores ≥1 (OR: 7.01; 95% CI: 1.83–26.94), gingivitis (OR: 17.65; 95% CI: 5.95–52.37), mean clinical attachment loss ≥2 mm (OR: 8.46; 95% CI: 3.47–20.63), and severe periodontitis (OR: 11.75; 95% CI: 3.89–35.49) with COVID‐19. Notably, these findings were more prevalent in the case group (Anand et al., 2021). Seventy participants (40 ± 9.8 years of age, 71.4% women) tested positive for SARS‐CoV‐2 RNA in the NASO/ORO samples of a study. According to Gomes et al. (2021), 13 dental biofilm samples tested positive (18.6%; 95% CI: 9.5, 27.7), indicating a higher virus load in NASO/ORO samples (*p* = .012) than those testing negative (Cq = 20.4 [6.1]). As per a previous study, men were more likely to be infected with COVID‐19, and advanced age was associated with a higher risk of periodontitis (Gupta et al., 2022). Higher severity of periodontitis led to 7.45 odds of requiring assisted ventilation, 36.52 odds of hospital admission, 14.58 odds of being deceased, and 4.42 odds of acquiring COVID‐19‐related pneumonia in this study. In another study, periodontitis was associated with COVID‐19‐related complications including death (OR: 8.81; 95% CI: 1.00–77.7), intensive care unit (ICU) admission (OR: 3.54; 95% CI: 1.39–9.05), and need for assisted ventilation (OR: 4.57; 95% CI: 1.19–17.4). Similarly, white blood cell, D‐dimer, and CRP levels were significantly higher in COVID‐19 patients with periodontitis in this study (Marouf et al., 2021).

## 8 DISCUSSION

This scoping review aimed to summarize the existing literature on the association between periodontitis and COVID‐19. The findings are inconclusive owing to a lack of evidence linking periodontal disease to an increased risk of COVID‐19 (Larvin et al., 2020). However, a case–control study discovered a link between periodontitis severity and COVID‐19 (Anand et al., 2021). This first‐of‐its‐kind study assessed all the potential periodontal markers and oral hygiene levels by directly examining patients with COVID‐19 (Anand et al., 2021). Another study discovered that periodontitis was associated with a higher risk of ICU admission, assisted ventilation, and death in patients with COVID‐19, as well as elevated biomarker levels that were associated with poor disease outcomes (Marouf et al., 2021).

### 8.1 Periodontal disease and its relationship with COVID‐19

Periodontal disease is characterized by progressive inflammation and destruction of periodontal tissues owing to the colonization by periodontopathogens. Moreover, this can lead to tooth loss and masticatory dysfunction in adults. The prevalence of periodontal disease increases with increasing age, with 70% of adults above 65 years of age experiencing periodontitis in the United States (Eke et al., 2016). Microbial dysbiosis has been identified in the pathogenesis of periodontitis. This dysfunction triggers a host immune defense against pathogenic bacteria through the stimulation of proinflammatory cytokines and cytokine‐mediated involvement of neutrophils at the site of infection (Pan et al., 2019). Periodontal bacteria have the potential to invade and populate other distant organs, and systemic dissemination is facilitated by damaging the gingival epithelial barrier and spreading through the bloodstream (Aquino‐Martinez & Hernandez‐Vigueras, 2021). Moreover, following translocation into the lower respiratory tract via the oral–lung aspiration axis, these bacteria might aggravate SARS‐CoV‐2‐induced severe lung infections by elevating the secretion of inflammatory cytokines, including interleukin‐6 (IL‐6).

Additionally, current research has established a link between oral microbiota and periodontal inflammation (Van Dyke et al., 2020). The primary role is played by the inflammatory continuum, followed by microbial specificity (pathogenicity). This concept is also consistent with the most recent periodontal disease classification (Caton et al., 2018), which now focuses on how the resolution of inflammation brings a change in microbial composition and restoration of microbiological homeostasis.

In addition, saliva appears to be another easy mode of transmission for COVID‐19, either via infected persons' nasal secretions, microdroplets, or coughs or via inhalation of salivary droplets into their own lower respiratory tract (Carrouel et al., 2021). However, this aspiration axis may not always promote the spread of harmful bacteria; rather, proinflammatory cytokines generated during periodontal inflammation disseminate into the circulation and contribute to the development of systemic diseases such as COVID‐19. Table 2 lists significant developments associated with periodontitis and COVID‐19.

**Table 2:** List of significant development in associating periodontitis and COVID‐19

Authors	Significant development
Takahashi et al. (2020)	An elevated level of cytokines in periodontitis metastasizes to the lungs to destroy epithelial cells exposing these cells to more viral attacks and damage.
Aquino‐Martinez et al. (2020)	LPS of *Porphyromonas gingivalis* disrupted the pulmonary epithelial integrity triggering senescence with a higher expression of vimentin which along with ACE2 drives more SARS‐CoV‐2 invasion.
Y. Li et al. (2019) and Mammen et al. (2020)	The oral microbiome colonizes distant organs, lungs by oral–lung aspiration axis, and influences pathogenicity by modifying mucosal surfaces.
Callender et al. (2020)	Seniors and those with comorbidities are more prone to SARS‐CoV‐2 infection.
Fang et al. (2019), Bouhaddou et al. (2020), Hirano and Murakami (2020), and Sahni and Gupta (2020)	SARS‐CoV‐2 entry triggers NF‐kB pathway and p38 MAPK activation by downregulation of ACE2, enhancing senescence and cytokine production, which are similar features observed in periodontitis.
Pan et al. (2019)	An unbalanced oral microbiome causes periodontitis and systemic disease by activating proinflammatory cytokines (IL‐1, IL‐7, IL‐10, IL‐17, IL‐8, TNF‐α, and MCP‐1) and neutrophils.
Bui et al. (2019)	Oral microbiome responsible for periodontitis and systemic diseases.
Venkataraman et al. (2015)	Lung microbiomes are more similar to oropharynx microbiomes than those in the nasopharynx or air.

Abbreviations: ACE2, angiotensin‐converting enzyme‐2; COVID‐19, coronavirus disease 2019; IL, interleukin; LPS, lipopolysaccharides; MAPK, mitogen‐activated protein kinase; MCP‐1, monocyte chemoattractant protein; NF‐kB, nuclear factor‐kB; SARS‐CoV‐2, severe acute respiratory syndrome coronavirus‐2; TNF‐α, tumor necrosis factor‐α.

### 8.2 Periodontal bacteria‐induced senescence driving SARS‐CoV‐2 invasion

Periodontal bacterial infection in the oral cavity accelerates senescence in distant organs through the oral–lung aspiration axis or inhalation of saliva. The most frequently implicated cells include lung epithelial, adipocyte precursor, and microglial cells. Prolonged contact with lipopolysaccharide (LPS), a component of *Porphyromonas gingivalis* cell wall, initiates cellular senescence followed by the recruitment of neutrophils, pro‐inflammatory cytokines, and phagocytic cells. Additionally, *P. gingivalis‐induced* periodontal inflammation stimulates the formation of reactive oxygen species (ROS), which results in the initiation and stabilization of DNA damage. As a result of increased ROS formation, the LPS‐induced oxidative stress results in DNA damage and the expression of pro‐and antiapoptotic proteins such as Bcl2.

These cascades of cellular reactions result in cell growth arrest due to the elevated expression of senescence‐associated β‐galactosidase, increased p53 levels, and enlarged cytoplasm undergoing apoptosis. Additionally, nuclear DNA damage has been associated with enhanced proinflammatory responses and innate immune activation, further propagating cellular senescence in the context of periodontal inflammation (Aquino‐Martinez et al., 2020). Incidentally, induced DNA damage results in dysfunctional cells in the pulmonary epithelium and degradation in the local microenvironment by increasing the expression of the transmembrane proteins vimentin and ACE2, which are required to facilitate an enhanced SARS‐CoV‐2 entry and viral replication (Aquino‐Martinez et al., 2020; Kim et al., 2016). Vimentin, a cytoskeletal filamentous protein that is highly expressed in senescent cells, acts as a coreceptor in addition to ACE2, contributing to the increased SARS‐CoV‐2 attachment to lung epithelial cells as ACE2 alone cannot drive efficient viral binding and entry (Yu et al., 2016).

### 8.3 Oral microbiota and influence on human lung diseases

The oral cavity houses the second highest number of microbes in the human body, including bacteria, archaea, fungi, and viruses. Previous studies examining microbial DNA from the calculus of ancient human remains have identified antibiotic‐resistant genes in opportunistic pathogens of the oral cavity (Warinner et al., 2014). These observations suggest that the oral cavity potentially acts as an ecological reservoir for several pathogens, subsequently triggering a set of local and systemic diseases. The main bacterial genera *Streptococcus*, *Prevotella*, *Neisseria*, *Corynebacterium*, *Leptotrichia*, *Veillonella*, *Fusobacterium*, and *Capnocytophaga* are abundant in the oral cavity. In contrast, healthy lungs are predominated by *Streptococcus*, *Fusobacterium*, *Pseudomonas*, *Veillonella*, *Prevotella*, and *Capnocytophaga*, which also colonize the oral cavity of healthy humans (Wypych et al., 2019).

Patients with ventilator‐assisted pneumonia (VAP) demonstrate the oropharyngeal colonization of pathogens underlying the pathophysiological mechanism behind VAP development (Bao et al., 2020). Further studies have provided evidence on bacterial pathogens (*Streptococcus oralis*, *Prevotella salivae*, and *Mycoplasma salivarium*) that populate the respiratory tract of healthy individuals asymptomatically, or predominating acellular bronchoalveolar lavage fluid of healthy individuals and patients with VAP, as identified by 16S rRNA gene sequencing (Bahrani‐Mougeot et al., 2007; Y. Li et al., 2019; Venkataraman et al., 2015). Periodontal microbes are also involved in the development of aspiration pneumonia (Ellen, 1992). Disruption of the oral microbiome has been shown to cause the dissemination of periodontal bacteria into the lungs and exacerbate existing systemic diseases (Jepsen et al., 2015; McCulloch et al., 2017). Moreover, the accumulation of senescent cells generated by aging, chronic inflammation, smoking, and bacterial infection accelerates the onset of these systemic diseases by attenuating tissue regeneration through stimulation and proinflammatory cytokines (Aquino‐Martinez et al., 2020). The oral cavity contains ACE2 receptors on all mucosal surfaces, particularly on the dorsum of the tongue; therefore, they could facilitate an entry into the cells of the SARS‐2 virus. Moreover, the periodontal sulcus or pocket functions as a reservoir for SARS‐2 (Xiang et al., 2020). This is particularly important in the case of the new Omicron variant because of its preference for the upper respiratory tract over the lungs/lower respiratory tract. Another important aspect is the role of salivary glands as reservoirs for the SARS‐2 virus. The virus found in saliva arises not only from the oral cavity/periodontal pockets but also from the salivary glands (J. Xu, Li, et al., 2020).

### 8.4 Lung inflammation and cytokine connection to SARS‐CoV‐2

The invasion of SARS‐CoV‐2 is mediated by the adherence to ACE2 receptors through the enzymatic cleavage of the SARS‐CoV‐2 spike protein involving furin‐TMPRSS2‐elastase, which is located on the host cell surface (Bestle et al., 2020; J. Wang, Jiang, et al., 2020). According to previous studies, ACE2 is highly expressed in type II alveolar epithelial cells, indicating that these cells serve as the primary targets for the viral attack, with 83% of cells positively stained for ACE2 receptors (Zhao et al., 2020). In the presence of oxidative stress and severe inflammation, ACE2 acts as an anti‐inflammatory and antioxidant factor, contributing to the protection and maintenance of cellular integrity by inhibiting the nuclear factor‐κB pathway (Fang et al., 2019). However, the entry of SARS‐CoV‐2 into the host cells, which is facilitated by the viral spike protein causes the virus–receptor complex internalization and ACE2 downregulation in infected cells (Zhang et al., 2020). As a result, the NF‐κB pathway is triggered, leading to p38/MAPK activation and an elevated level of cytokine release, including IL‐6 and tumor necrosis factor‐α (TNF‐α) (Bouhaddou et al., 2020; Fang et al., 2019; Hirano & Murakami, 2020).

Moreover, microbial pathogens and nuclear DNA damage converge to activate NF‐κB, mediated by Toll‐like receptor 4 and ataxia telangiectasia mutated, which primarily sense double‐stranded DNA breaks within the nucleus (Aquino‐Martinez et al., 2020). Activated p38 is involved in cell growth arrest and promotes cellular senescence by influencing the senescence‐stimulating factors. Moreover, its inhibition by SB203580 is associated with a delayed onset of senescence. It has been speculated that the accumulation of *P. gingivalis‐derived* LPS exacerbated the senescence in lung tissues, which increased the likelihood of precipitating a greater level of SARS‐CoV‐2 infection and enhancing viral replication (Kim et al., 2016). However, further investigation is required to validate this hypothesis.

**Figure 1:**
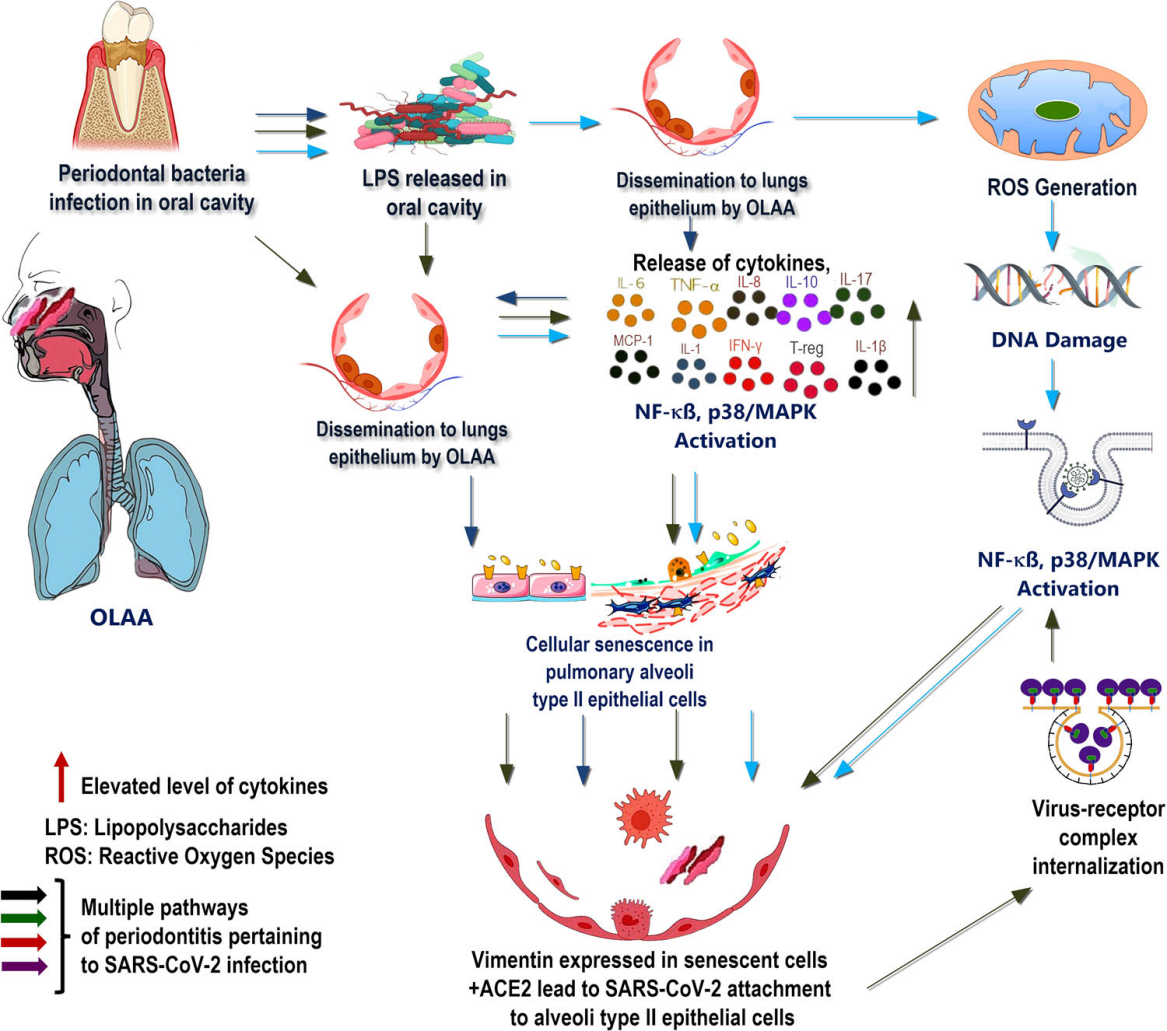
Multiple pathways of periodontitis contribute to cellular senescence in the lungs epithelium and influencing the risk of SARS‐CoV‐2 infection to host cells. ACE2, angiotensin‐converting enzyme‐2; IFN, interferon; IL, interleukin; LPS, lipopolysaccharide; MAPK, mitogen‐activated protein kinase; MCP, monocyte chemoattractant protein; NF, nuclear factor; OLAA, oral–lung aspiration axis; ROS, reactive oxygen species; SARS‐COV‐2, severe acute respiratory syndrome coronavirus‐2; TNF, tumor necrosis factor; T‐regs, regulatory T cells.

Previous experiments demonstrating a relationship between a healthy oral microbiome and the development of systemic diseases, including COVID‐19, have attempted to elucidate the association between periodontitis and COVID‐19. In particular, *P. gingivalis* increases its virulence by evading the host immune surveillance through the delayed infiltration of neutrophils and inhibition of IL‐8 secretion at the site of infection (Darveau et al., 1998). The lack of IL‐8 is associated with the overgrowth of these pathogens and increased SARS‐CoV‐2 invasion because of the disrupted cellular integrity in the pulmonary alveolar epithelial lining. Elevated cytokine levels further accelerate this disruption by disseminating periodontopathogens to the oral cavity and lungs. Consequently, a high expression profile involving proinflammatory cytokines, IL‐1, IL‐7, IL‐10, IL‐17, IL‐8, TNF‐α, and monocyte chemoattractant protein‐1, were detected both in patients with periodontitis and those with COVID‐19 (Sahni & Gupta, 2020) (Figure 1). In addition, IL‐17 has been identified as a senescence stimulant that exacerbates lung inflammation and could serve as a plausible biomarker for COVID‐19. These observations confirmed that the cytokines disseminated to other distant organs, including the lungs, through the bloodstream or oral‐lung aspiration axis, induce damage to the lung epithelial lining, and create favorable conditions for severe SARS‐CoV‐2 infection.

### 8.5 T‐cell responses against periodontitis and its connection with COVID‐19

Regulatory T cells (T‐regs) and T helper (Th) cells protect against bacterial infection and periodontal invasion in the lungs. According to previous research, bacteria and their metabolites, such as LPS and short‐chain fatty acids translocated from the oral cavity into the lungs, contribute to the development of the T‐reg response. Previous exposure to periodontopathogens (*Prevotella* sp.) and their metabolites are associated with the inhibited production of interferon‐γ and IL‐17A, which confer higher susceptibility to tuberculosis and other respiratory diseases, including severe COVID‐19 disease (Segal et al., 2017). A study involving 1099 patients in China demonstrated that patients with severe COVID‐19 presented with peripheral lymphocytopenia and hyperactivated CD4+ and CD8+ T cells. A dysregulated T‐cell response is accelerated by elevated proinflammatory CCR6+ Th17 in CD4+ T cells and a high concentration of cytotoxic granules in CD8+ T cells. These observations suggest that, in addition to chronic periodontal inflammation, hyperactivated and reduced levels of CD4+ and CD8+ T cells are implicated in immune injury and the onset of cytokine storm in the lungs and other vital organs of patients with COVID‐19, which are associated with severe COVID‐19 cases, COVID‐19‐induced pneumonia, and death.

### 8.6 Host factors and periodontal therapy

Middle‐aged and older adults with periodontitis and other comorbidities, or those harboring an unhealthy oral microbiome with impaired immune functions, are more susceptible to SARS‐CoV‐2 infection and its associated complications, even after eliminating the virus from the system (Chen et al., 2020). Cancer malignancies, chronic obstructive pulmonary disease, diabetes, and hypertension appear to be driving factors for promoting viral entry and evasion of host immune defense, leading to severe disease progression and eventually death (Guan et al., 2020). Since microbial dysbiosis, bacterial superinfection, and host hyperresponsiveness play vital roles in the severity of COVID‐19, emphasis should be placed on periodontal maintenance (Sukumar & Tadepalli, 2021). Interestingly, evidence indicates a potential role of Galectin‐3 mediated increased immune response and increased viral attachment in the association between periodontal disease and COVID‐19. Moreover, an area in the coronavirus spike protein depicts a morphology that is highly similar to that of Galectin‐3. Therefore, the maintenance of a healthy periodontal status appears critical in SARS‐CoV‐2 infection for control during the COVID‐19 pandemic (Kara et al., 2020; Larvin et al., 2020).

In addition, a strong correlation between the severity of COVID‐19 and type 2 diabetes in patients with periodontitis was recently reported, showing that the number of ACE2 receptors was prominently higher in those with periodontal disease and diabetes than in those with periodontal disease alone (Casillas Santana et al., 2021). In other words, diabetes and periodontal disease significantly contributed to the increase in the number of ACE2 receptors among individuals, thereby enhancing the susceptibility to SARS‐CoV‐2 infection and subsequently accelerating the disease prognosis. However, further clinical studies are needed to explore whether patients with COVID‐19 might benefit from periodontal treatment, as it could reduce the severity of complications (Shamsoddin, 2021).

## 9 CONCLUSION AND FUTURE PERSPECTIVE

The current COVID‐19 pandemic has heightened concerns about disease manifestation heterogeneity across infected individuals, particularly older adults with comorbidities. A small number of individuals develop acute respiratory distress and hypoxia as a result of severe exudation and formation of hyaline membranes in the alveolar spaces. COVID‐19‐related research involving survivors and affected individuals have examined the pathogenesis of disease onset and progression, focusing on COVID‐19‐induced pneumonia, cytokine storm, septic shock, and multiorgan failure, which resulted in the untimely death of cases and a rising death toll worldwide. To gain a better understanding of the mechanism underlying the increased disease susceptibility, researchers have established a link between COVID‐19 development and the presence of comorbidities such as COPD, aging, diabetes, hypertension, cardiac problems, renal problems, and altered gut and oral microbiomes. The impact of aging and other comorbidities has been previously demonstrated to be associated with neurological diseases, cardiovascular diseases, risk of cerebrovascular accidents, and renal problems.

Senescence is induced in cells in the production of ROS and irreversible nuclear DNA damage caused by the activation of the NF‐κB and p38/MAPK pathways. This scoping review describes the mechanism by which the disrupted oral microbiota induce senescence in the pulmonary epithelial lining, resulting in increased SARS‐CoV‐2 entrance into type II alveolar epithelial cells, where ACE2 and vimentin promote the expression of a filamentous protein that acts as a coreceptor for more viral invasion. It also highlights the contribution of *P. gingivalis*‐derived LPS and elevated levels of cytokines such as IL‐1, IL‐7, IL‐10, IL‐17, IL‐8, TNF‐α, and IL‐6 in disseminating lungs along with other distant organs through the oral‐lung aspiration axis (Figure 1) or blood flow and inhalation of saliva to induce further viral infection and its associated complications. Additionally, the presence of *P. gingivalis*, a critical periodontal bacterium, impairs immune surveillance by impairing the neutrophil recruitment and blocking the production of IL‐8 at the infection site via chemokine paralysis. Furthermore, these were implicated in increased susceptibility to higher SARS‐CoV‐2 infection and viral replication‐propagating disease onset. Therefore, exacerbated immune responses escalate the cellular senescence involving the gingival epithelial barrier and pulmonary alveolar epithelial cells, in addition to chronic bacterial infection, thus demonstrating an association between periodontitis and the development of COVID‐19.

## AUTHOR CONTRIBUTIONS

Both Arunibha Ghosh and Betsy Joseph conceived the idea of a review paper to highlight the mechanism underlying the connection between periodontitis and COVID‐19. Arunibha Ghosh drafted the manuscript. Betsy Joseph, Anil Sukumaran, and Arunibha Ghosh revised the manuscript. All the authors (Arunibha Ghosh, Betsy Joseph, and Anil Sukumaran) approved the final draft of the manuscript.

## CONFLICTS OF INTEREST

The authors declare no conflicts of interest.

## Data Availability

The data sets used and/or analyzed during the current study are available from the corresponding author on reasonable request.
